# Aquincola agrisoli sp. nov., isolated from rhizospheric soil of eggplant and in silico genome mining for the prediction of biosynthetic gene clusters

**DOI:** 10.1099/ijsem.0.006355

**Published:** 2024-04-29

**Authors:** Md. Amdadul Huq, Md. Shahedur Rahman, M. Mizanur Rahman

**Affiliations:** 1 Department of Food and Nutrition, College of Biotechnology and Natural Resource, Chung-Ang University, Anseong-si, Gyeonggi-do, 17546, Republic of Korea; 2 Department of Genetic Engineering and Biotechnology, Jashore University of Science and Technology, Jashore 7408, Bangladesh; 3 Department of Biotechnology and Genetic Engineering, Faculty of Biological Science, Islamic University, Kushtia-7003, Bangladesh

**Keywords:** *Aquincola agrisoli*, digital DNA–DNA hybridisation, genome sequence, *in silico *genome mining, secondary metabolites

## Abstract

A Gram-stain negative, aerobic, rod-shaped, motile and flagellated novel bacterial strain, designated MAHUQ-54^T^, was isolated from the rhizospheric soil of eggplant. The colonies were observed to be light pink coloured, smooth, spherical and 0.2–0.6 mm in diameter when grown on R2A agar medium for 2 days. MAHUQ-54^T^ was able to grow at 15–40 °C, at pH 5.5–9.0 and in the presence of 0–0.5 % NaCl (w/v). The strain gave positive results for both catalase and oxidase tests. The strain was positive for hydrolysis of l-tyrosine, urea, Tween 20 and Tween 80. On the basis of the results of 16S rRNA gene sequence comparisons, the isolate was identified as a member of the genus *Aquincola* and is closely related to *Aquincola tertiaricarbonis* L10^T^ (98.8 % sequence similarity) and *Leptothrix mobilis* Feox-1^T^ (98.2 %). MAHUQ-54^T^ has a draft genome size of 5 994 516 bp (60 contigs), annotated with 5348 protein-coding genes, 45 tRNA and 5 rRNA genes. The average nucleotide identity (ANI) and digital DNA–DNA hybridisation (dDDH) values between MAHUQ-54^T^ and its closest phylogenetic neighbours were 75.8–83.3 and 20.8–25.3 %, respectively. *In silico* genome mining revealed that MAHUQ-54^T^ has a significant potential for the production of novel natural products in the future. The genomic DNA G+C content was determined to be 70.4 %. The predominant isoprenoid quinone was ubiquinone-8. The major fatty acids were identified as C_16  :  0_, summed feature 3 (comprising C_16  :  1_ω7*c* and/or C_16  :  1_ω6*c*) and summed feature 8 (comprising C_18  :  1_ω7*c* and/or C_18  :  1_ω6*c*). On the basis of dDDH, ANI value, genotypic analysis, chemotaxonomic and physiological data, strain MAHUQ-54^T^ represents a novel species within the genus *Aquincola*, for which the name *Aquincola agrisoli* sp. nov. is proposed, with MAHUQ-54^T^ (=KACC 22001^T^ = CGMCC 1.18515^T^) as the type strain.

## Introduction

The genus *Aquincola*, first described by Lechner *et al*. [1], is a member of the *Rubrivivax– Roseateles–Leptothrix– Ideonella– Aquabacterium* branch of the class *Betaproteobacteria* [2]. The genus *Aquincola* presently comprises three species with validly published names: *Aquincola tertiaricarbonis*, isolated from methyl *tert*-butyl ether (MTBE)-contaminated groundwater in Germany [1] and a wastewater plant in France [3], *Aquincola amnicola*, isolated from a freshwater river in Taiwan [4] and *Aquincola rivuli*, isolated from a freshwater stream in Taiwan [5]. Cells are Gram-stain-negative, obligately aerobic, non-spore-forming, rod-shaped, motile by means of a single polar flagellum and catalase- and oxidase-positive. Chemotaxonomically, cells possess Q-8 as the major respiratory quinone, summed feature 3 (comprising C_16 : 1_ω7*c* and/or C_16 : 1_ω6*c*) and C_16 : 0_ as the predominant fatty acids and DNA G+C contents between 69.0 and 70.7 mol% [1,5]. In the present study, we report a Gram-stain-negative bacterium, MAHUQ-54^T^, which was isolated during the characterisation of the bacterial diversity in the rhizospheric soil of eggplant. Phylogenetic analyses based on 16S rRNA gene and genome sequences and polyphasic characterisation revealed that this isolate represented a member of the genus *Aquincola* and a novel species. The purpose of this study is to clarify the taxonomic position of MAHUQ-54^T^ in detail on the basis of phenotypic characteristics and the results of chemotaxonomic and genotypic analysis. Moreover, the genome sequence of MAHUQ-54^T^ was analysed for the presence of putative natural product biosynthetic gene clusters (BGCs). The availability of whole-genome sequences and synthetic biology-inspired tools/approaches make it possible to utilise these BGCs to develop new chemicals with new structures, new activity and new targets [6]. Our data revealed that MAHUQ-54^T^ contains BGCs, indicating the potential capability to produce new chemicals with biological activity.

## Isolation and cultivation

During the investigations of bacterial biodiversity, a novel bacterium, designated MAHUQ-54^T^, was isolated from a sample of rhizospheric soil of an eggplant located in Magura, Bangladesh. A quantity (1 g) of soil sample was suspended in 9 ml of sterile 0.85 % (w/v) NaCl solution. The suspension was serially diluted up to a 10^−6^ dilution and 200 µl suspension was spread onto Reasoner’s 2A (R2A) agar plates (MB cell). The plates were incubated at 30 °C for 3 days. Single colonies were purified by repeated streaking on fresh R2A agar plates and preserved as a suspension in R2A broth containing glycerol (25 %, v/v) at −80 °C. On the basis of the results of 16S rRNA gene sequence analysis, MAHUQ-54^T^ was shown to be a novel bacterium and was selected for detailed taxonomic studies. MAHUQ-54^T^ has been deposited to the Korean Agricultural Culture Collection (KACC) and China General Microbiological Culture Collection Centre (CGMCC).

## 16s rRNA gene, genome and phylogenetic analysis

Extraction of the genomic DNA was achieved using a commercial genomic DNA extraction kit (Solgent). The 16S rRNA gene was amplified from the chromosomal DNA with the universal bacterial primer pair 27F (5′-AGAGTTTGATCCTGGCTCAG-3′) and 1492R (5′-GGTTACCTTGTTACGACTT-3′) [7] and the purified PCR products were sequenced by Solgent (Daejeon, Republic of Korea). The 16S rRNA gene sequences of related taxa were obtained from the GenBank database (http://blast.ncbi.nlm.nih.gov/Blast.cgi) and EzBioCloud server (https://www.ezbiocloud.net) [8]. The multiple sequence alignments were performed by using the clustal_x programme [9]. Gaps were edited using the BioEdit programme [10]. The evolutionary distances were calculated using the Kimura two-parameter model [11]. The phylogenetic trees were reconstructed based on 16S rRNA gene sequences using the neighbor-joining (NJ) [12], maximum-likelihood (ML) and maximum-parsimony (MP) algorithms in the mega 7.0 programme [13], with bootstrap values based on 1000 replications. The phylogenetic trees were also reconstructed using whole-genome sequences based on multi-locus sequence analysis (MLSA; https://automlst.ziemertlab.com/analyze) [14]. The draft genome sequence of MAHUQ-54^T^ was determined using an HiSeq X Ten (Illumina) and was assembled using the SOAPdenovo v. 3.10.1 *de novo* assembler. The genome annotation was performed using the NCBI prokaryotic genome annotation pipeline (PGAP). To estimate the degree of pairwise relatedness between MAHUQ-54^T^ and the closest reference strains, blast-based average nucleotide identity (ANI) was calculated as described previously [15]. While the digital DNA–DNA hybridisation (dDDH) value was determined using the genome-to-genome distance calculator (http://ggdc.dsmz.de/ggdc.php) according to the methods of Meier-Kolthoff *et al.* [16].

**Fig. 1.**
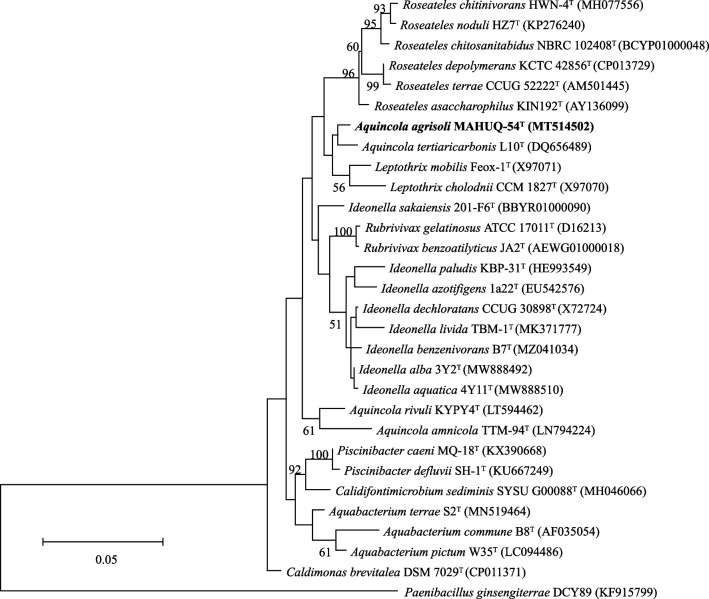
The maximum-likelihood (ML) tree based on the results of 16S rRNA gene sequence analysis showing the position of *Aquincola agrisoli* MAHUQ-54^T^ and related species. Bootstrap values less than 50 % based on 1000 replications are not shown at branching points. '*Paenibacillus ginsengiterrae*' DCY89 was used as an outgroup. Bar, 0.05 substitutions per nucleotide position.

According to the results of EzBioCloud server analysis, 16S rRNA gene sequences indicated that the closest relations of strain MAHUQ-54^T^ were *Aquincola tertiaricarbonis* L10^T^ (98.8 %) and *Leptothrix mobilis* Feox-1^T^ (98.2 %). Similarities with all other strains were less than 97.6 %. The 16S rRNA gene sequence of MAHUQ-54^T^ is a continuous stretch of 1448 bp (NCBI GenBank accession number MT514502). The relationship between MAHUQ-54^T^ and other species was supported by the topology of the phylogenetic trees (Fig. 1;Figs S1 and S2, available in the online version of this article). The ML tree indicated that MAHUQ-54^T^ clustered within the genus *Aquincola* and formed a monophyletic clade with *Aquincola tertiaricarbonis* L10^T^ (Fig. 1). The ML tree was also supported by the trees created using the NJ and MP algorithms (Figs S1 and S2) with high bootstrap values. Moreover, the phylogenetic tree that was reconstructed from MLSA of whole-genome sequences indicated that MAHUQ-54^T^ is clustered with the members of genus *Aquincola* and formed a monophyletic clade with *Aquincola tertiaricarbonis* L10^T^ (Fig. S3). The results of phylogenetic analysis indicated that MAHUQ-54^T^ is clearly grouped within the genus *Aquincola.* The draft genome sequence of MAHUQ-54^T^ yielded a genome of 6.0 Mb in length after assembly, producing 60 contigs with an N_50_ value of 359 003. The total genome size is 5 994 516 bp. Gene prediction allowed the annotation of 5348 protein-coding genes with 45 tRNA and 5 rRNA genes. The genomic DNA G+C content of MAHUQ-54^T^, directly calculated from its genome sequence, was determined to be 70.4 % which is in the range of the type strains of species of the genus *Aquincola* [1,5]. The genome sequence features of MAHUQ-54^T^ are listed in Table S1. The ANI values between MAHUQ-54^T^ and phylogenetically close neighbours *Aquincola tertiaricarbonis* L10^T^ and *Leptothrix mobilis* Feox-1^T^ were 83.35 and 75.89 %, respectively (Table S2). The dDDH values between MAHUQ-54^T^ and *Aquincola tertiaricarbonis* L10^T^ and *Leptothrix mobilis* Feox-1^T^ were 25.3 and 20.8 %, respectively (Table S2). These ANI values and dDDH values are well below the species thresholds of 95–96 and 70 %, respectively, indicating that MAHUQ-54^T^ represents a novel species [17,19]. On the basis of dDDH results, ANI values and the results of phylogenetic analysis, it is evident that the isolated strain represents a novel species of the genus *Aquincola*.

## Comparative genomic studies

For a whole-genome-based taxonomic analysis, the genome sequence data were uploaded to the Type (Strain) Genome Server (TYGS), a free bioinformatics platform accessible at https://tygs.dsmz.de (accessed 15 February 2024). The Genome blast Distance Phylogeny (GBDP) approach was also used to calculate dDDH values and to reconstruct phylogenetic trees using TYGS [20,21]. GBDP phylogenetic trees were reconstructed using both 16S rRNA sequences and whole-genome sequences. CGView (http://cgview.ca/) was used to generate a graphical representation of the blast result comparison of the available genomes with the genome of MAHUQ-54^T^. Taxonomic and functional research on microorganisms has increasingly relied upon genome-based data and methods [22]. The distribution of genes in the genome of MAHUQ-54^T^ and the most closely related reference strain *Aquincola tertiaricarbonis* L10^T^ was investigated using the Rapid Annotation using Subsystems Technology (RAST) server [23].

**Fig. 2.**
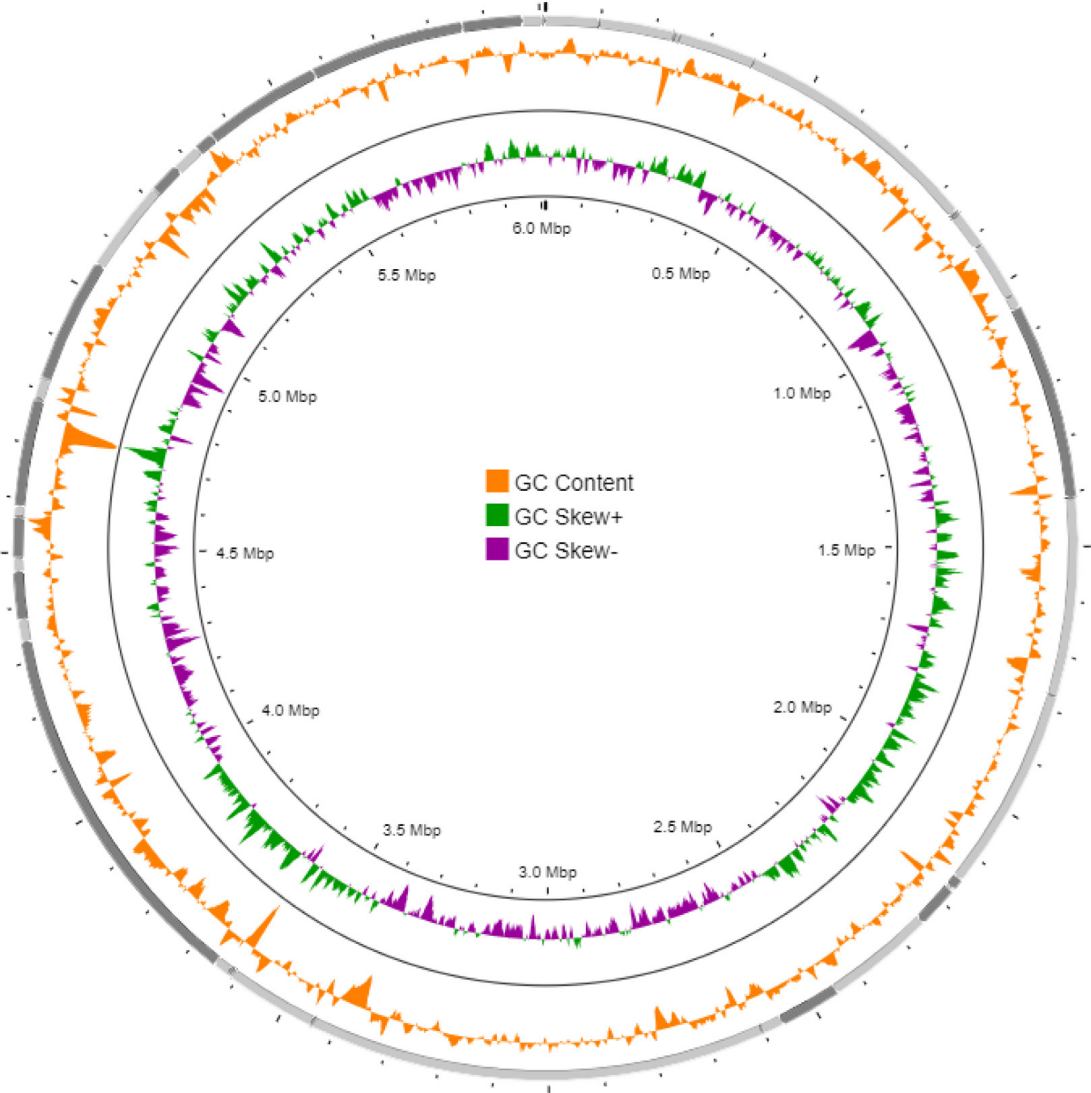
Schematic representation of the circular chromosome of MAHUQ-54^T^, created using the CGView server (http://cgview. ca/). The outermost circle displays the contigs while the middle circle displays the DNA G+C content plot and the innermost circle displays the G+C skew. To indicate genome sizes inside and outside, the ruler was used in the chromosome map.

Using the GBDP method and tree builder service, the phylogenetic trees of MAHUQ-54^T^, using its 16S rRNA gene sequence and whole genome sequence, were created. The GBDP phylogenetic tree reconstructed by using 16S rRNA indicated that MAHUQ-54^T^ clustered with the members of the genus *Aquincola* and formed a monophyletic clade with *Aquincola tertiaricarbonis* L10^T^ (Fig. S4). Similarly, the GBDP phylogenetic tree reconstructed by using the whole genome sequence indicated that MAHUQ-54^T^ clustered with the members of the genus *Aquincola* and formed a monophyletic clade with *Aquincola tertiaricarbonis* L10^T^ (Fig. S5). In all, the 16S rRNA-based GBDP phylogenetic tree, genome-based GBDP phylogenetic tree and whole genome alignment and the results of comparative genome analysis (pairwise comparisons of user genomes vs type strain genomes, Table S3) indicated that MAHUQ-54^T^ represented a novel species belonging to the genus *Aquincola*. Fig. 2 shows the circular chromosomes based on the genome sequence of MAHUQ-54^T^ generated using CGView server (http://cgview.ca/), which is a web-based tool for comparative genomics analysis on circular genomes [24]. The RAST functional annotations of the draft genome of MAHUQ-54^T^ indicated that 201 of the genes were involved in protein metabolism, 337 genes were associated with the metabolism of amino acids and derivatives, 82 genes were involved in DNA metabolism, 280 genes were linked with carbohydrate metabolism and 187 genes were involved in the metabolism of vitamins, cofactors and pigments. Moreover, the genome of MAHUQ-54^T^ revealed 81 gene clusters for stress response and 116 genes for respiration (Table S4). The genome of MAHUQ-54^T^ has 36 genes for motility and chemotaxis (Table S4). The presence of genes for flagellar motility and the presence of flagella (Fig. S6) indicated that the phenotypic and genomic results are consistent with each other. RAST functional analysis revealed that the genome of the most closely related type strain *Aquincola tertiaricarbonis* L10^T^ contains the same genes but there were quantitative differences (Table S4). For example, the genome of MAHUQ-54^T^ contains 39 genes which are responsible for virulence, disease and defence but the genome of *Aquincola tertiaricarbonis* L10^T^ contains 36 genes in this category. Similarly, the genome of MAHUQ-54^T^ contains 36 genes that are responsible for regulation and cell signalling but type strain *Aquincola tertiaricarbonis* L10^T^ contains 33 genes in this category (Table S4).

## Secondary metabolite biosynthetic gene cluster prediction

As a main approach for finding and annotating genes in BGCs across the genome, antiSMASH 7 [25] combined with ClusterBlast, ActiveSiteFinder, Cluster PFam analysis and SubClusterBlast [25] was used for the discovery of BGCs in the genome of *Aquincola agrisoli* MAHUQ-54^T^ for secondary metabolites.

**Fig. 3.**
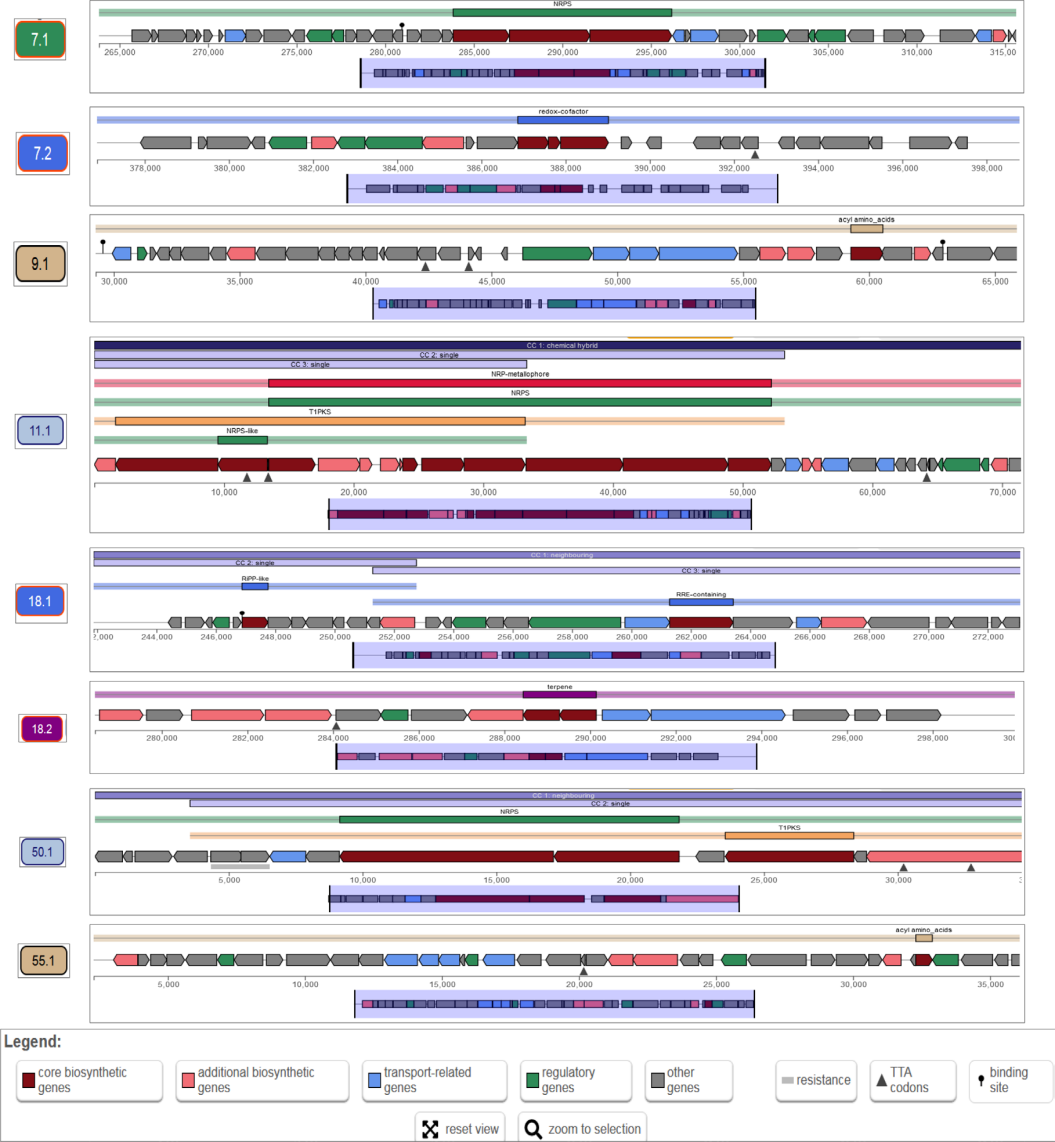
Localisation of secondary metabolite clusters in the genome of *Aquincola agrisoli* MAHUQ-54^T^.

Using antiSMASH 7.0, we found several BGCs in the genome of the novel strain *Aquincola agrisoli* MAHUQ-54^T^ for different secondary metabolites. Through prediction using antiSMASH 7.0, eight BGCs were discovered in the genome of *Aquincola agrisoli* MAHUQ-54^T^ (Table 1 and Fig. 3). The BGC types include those for nonribosomal peptide-synthetase (NRPS), redox-cofactor, acyl amino acids, non-ribosomal peptide (NRP)-metallophore, NRPS, type I polyketide synthases (T1PKS), NRPS-like, ribosomally synthesised and post-translationally modified peptide (RiPP)-like, RiPP recognition element (RRE)-containing, terpene, NRPS, T1PKS and acyl amino acids were discovered in the genome of MAHUQ-54^T^ (Table 1 and Fig. 3). All of these BGCs exhibited just a low degree of similarity or resemblance to previously identified BGCs, implying that MAHUQ-54^T^ has a significant potential for the production of novel natural products in the future.

**Table 1.** The analysis of biosynthetic pathways in *Aquincola agrisoli* MAHUQ-54^T^ by antiSMASH 7.0

Cluster serial number	Region	Type	From	To	Most similar known cluster	Similarity
1	7.1	NRPS	263837	316176		
2	7.2	Redox-cofactor	376866	399019		
3	9.1	Acyl amino acids	29 279	66 261	Teixobactin, NRP	30 %
4	11.1	NRP-metallophore, NRPS, T1PKS, NRPS-like	1	72 215	Fabrubactin A/Fabrubactin B, NRP+Polyketide	14 %
5	18.1	RiPP-like, RRE-containing	241883	273430		
6	18.2	Terpene	278440	300145		
7	50.1	NRPS, T1PKS	1	35 001		
8	55.1	Acyl amino acids	2291	36 440		

## Physiology and chemotaxonomy

The Gram-stain reaction was determined using a bioMérieux Gram stain kit according to the manufacturer’s instructions. Growth was tested using several bacterial media, such as nutrient agar (NA) (Difco), tryptone soya agar (TSA) (Oxoid), R2A agar, Luria–Bertani (LB) agar (Oxoid) and MacConkey agar (Oxoid) at 30 °C. Growth at different temperatures (4, 10, 15, 20, 25, 28, 30, 35, 40 and 45 °C) and at various pH conditions (pH 3.0–10.0, at intervals of 0.5 pH units) was assessed in the R2A broth after 7 days of incubation at 30 °C. The effect of pH on growth was tested using two buffers (final concentration, 100 mM): acetate buffer was used for pH 3.0–6.5 and phosphate buffer was used for pH 7.0–10.0. Salt tolerance was tested in R2A broth supplemented with 0–5.0 % (w/v) NaCl (at 0.5 % intervals) after 7 days of incubation at 30 °C. Growth was estimated by monitoring the OD_600_. To check anaerobic growth, MAHUQ-54^T^ was cultured on R2A plates and incubated in a closed chamber with an AnaeroGen kit (Thermo Scientific) which was used for the generation of an anaerobic atmosphere for 14 days at 30 °C [26]. Cell morphology was observed at ×11 000 magnification with a transmission electron microscope (JEM1010; JEOL) using cells grown for 3 days at 30 °C on R2A agar. Motility was assayed on sulphide–indole motility medium (Difco) [27]. Production of flexirubin-type pigments was determined using the procedures as outlined by Fautz and Reichenbach [28]. Catalase activity was determined using bubble production in 3 % (v/v) H_2_O_2_ and oxidase activity was determined using 1 % (w/v) *N*, *N*, *N*′, *N*′-tetramethyl-1, 4-phenylenediamine reagent as described by Huq [29]. Hydrolysis of the following substrates was tested using R2A agar as the basal medium: casein (2.0 % skim milk, Oxoid), 1.0 % starch (Difco), 0.1 % aesculin (0.02 % ferric citrate, Difco), Tween 80 (0.01 % CaCl_2_.2H_2_O and 1.0 % Tween 80, Sigma), Tween 20 (0.01 % CaCl_2_.2H_2_O and 1.0 % Tween 20, Sigma), 0.5 % l-tyrosine (Sigma), 12.0 % gelatin (Sigma) and DNA (DNase agar, Scharlau), DNase activity was revealed by flooding the plates with 1 M HCl. Activities were evaluated after 7 days of incubation at 30 °C. For comparative study, the reference strain *Aquincola tertiaricarbonis* L10^T^ was included and tested using the same laboratory conditions. Carbon-source utilisation and constitutive enzyme activities of MAHUQ-54^T^ and the reference strain were tested using API ZYM and API 20NE test kits (bioMérieux), according to the manufacturer’s instructions.

MAHUQ-54^T^ was aerobic, Gram-stain-negative and catalase- and oxidase-positive and had motile, flagellated rod-shaped cells (0.5–1.1 µm wide and 1.2–2.7 µm long) (Fig. S6). The colonies were observed to be light pink coloured, smooth, spherical and 0.2–0.6 mm in diameter when grown on R2A agar medium for 2 days. Growth occurred at 15–40 °C (optimum 30–35 °C) and at pH 5.5–9.0 (optimum pH 7.0). In R2A broth medium, growth occurred in the presence of 0–0.5 % (w/v) NaCl (optimum 0 %). MAHUQ-54^T^ was positive for the following enzyme activities: alkaline phosphatase, acid phosphatase, leucine arylamidase, valine arylamidase, cysteine arylamidase, trypsin, naphthol-AS-BI-phosphohydrolase, *N*-acetyl-β-glucosaminidase, esterase (C4), esterase lipase (C8), α-chymotrypsin, lipase (C14) and β-glucuronidase. Phenotypic and biochemical examinations revealed that MAHUQ-54^T^ shared several traits in common with its closest relative. However, there are some morphological, physiological and biochemical differences between MAHUQ-54^T^ and phylogenetically closely related species that differentiate MAHUQ-54^T^ from related type strains (Table 2). For example, MAHUQ-54^T^ can be easily differentiated from the phylogenetically closest species *Aquincola tertiaricarbonis* L10^T^ by the colony colour, nitrate reduction ability, arginine dihydrolase activity, urea degrading ability, inability to assimilate d-glucose, *N*-acetyl-glucosamine, maltose and d-mannose and different growth conditions. The physiological, morphological and biochemical characteristics of MAHUQ-54^T^ and the most closely related reference strain are summarised in Table 2 and the species description.

**Table 2.** The biochemical and physiological characteristics of MAHUQ-54^T^ and the most closely related reference strain Strains: 1, *Aquincola agrisoli* MAHUQ-54^T^; 2, *Aquincola tertiaricarbonis* L10^T^. All data were obtained during this study, except where indicated . All strains are rod shaped, aerobic, flagellated and motile. All strains are positive for catalase, oxidase, alkaline phosphatase, acid phosphatase, leucine arylamidase, valine arylamidase, cystine arylamidase, β-glucuronidase, hydrolysis of Tween 80 and Tween 20 and assimilation of d-mannitol, gluconate and malic acid. All strains are negative for indole production, fermentation of glucose, α-mannosidase, α-galactosidase, β-galactosidase, α-fucosidase, hydrolysis of starch, DNA, aesculin and gelatin and assimilation of l-arabinose, capric acid and triosodium citrate. +, Positive; w, weakly positive; −, negative.

Characteristics	1	2
Isolation source	Rhizospheric soil	Contaminated groundwater
Cell size (um)	0.5–1.1×1.2–2.7	0.8–1.1×1.2–2.3*
Colony colour	Light pink	White
Reduction of nitrate (API 20 NE)	+	−
Arginine dihydrolase	+	−
Growth temperature (Optimum, °C)	15–40 (30-35)	10–40 (25–30)*
Growth pH (Optimum)	5.5–9.0 (7.0)	5.5–8 (6.5)*
NaCl tolerance (Optimum, %)	0–0.5 (0)	0–1.0 (0)*
**Hydrolysis of:**		
Casein	−	+
Urea (API 20 NE)	+	−
**Enzyme activity (API ZYM):**		
Esterase (C4)	w	+
Esterase lipase (C8)	w	+
Lipase (C14)	w	+
Trypsin	w	−
α-Chymotrypsin	+	−
Naphthol-AS-BI-phosphohydrolase	w	+
*N*-acetyl-β-glucosaminidase	w	−
α-Glucosidase	−	+
β-Glucosidase	−	+
**Assimilation of (API 20 NE):**		
d-glucose	−	+
*N*-acetyl-glucosamine	−	+
Maltose	−	+
d-mannose	−	+
Adipic acid	w	−
Phenylacetic acid	+	−
DNA G+C content (%)	70.4	70.2

*Data from Sheu *et al.* [5].

For fatty acid methyl ester analysis, cells of MAHUQ-54^T^ and the reference strain were harvested from R2A agar plates after incubation for 3 days at 30 °C and fatty acids were determined as described by Sasser [30]. The isoprenoid quinones of MAHUQ-54^T^ were extracted from freeze-dried cell material. Quinones were extracted with chloroform/methanol (2 : 1, v/v) and extracts were evaporated under vacuum. The crude hexane–quinone solution was purified using Sep-Pak Vac silica cartridges (Waters) and subsequently analysed using a RP-HPLC system (Alliance 2690 system; Waters) [column; SunFire C18 (4.6×250 mm × 5 µm), solvent; methanol/2-propanol (7:5, v/v), flow rate; 1.0 ml min^−1^] [31,32].

The fatty acid profiles of MAHUQ-54^T^ and the related type strain *Aquincola tertiaricarbonis* L10^T^ are shown in Table 3. The major cellular fatty acids of MAHUQ-54^T^ were identified as C_16  :  0_ (25.0 %), summed feature 3 (C_16  :  1_ω7*c* and/or C_16  :  1_ω6*c*, 39.7 %) and summed feature 8 (comprising C_18  :  1_ω7*c* and/or C_18  :  1_ω6*c*, 17.2 %). MAHUQ-54^T^ showed a similar major fatty acid composition to the closely related type strain, but there were quantitative differences when cultivated under the same conditions (Table 3). The major respiratory quinone of MAHUQ-54^T^ was identified as ubiquinone-8 (Q-8), which is one of the characteristics of members of the genus *Aquincola* [1,5].

**Table 3.** Fatty acid profiles of MAHUQ-54^T^ and the most closely related reference strain Strains: 1, *Aquincola agrisoli* MAHUQ-54^T^; 2, *Aquincola tertiaricarbonis* L10^T^. All data were obtained during this study. Fatty acid percentages amounting to less than 1 % of the total fatty acids in all strains were not included in the table. tr, Trace (less than 1.0 %); nd, not detected.

Fatty acid	1	2
C_10 : 0_ 3OH	2.2	2.7
C_12 : 0_	3.3	4.5
C_14 : 0_	1.1	1.8
C_15 : 1_ω*6c*	1.7	1.3
C_16 : 0_	25.0	30.9
C_17 : 0_	1.9	1.1
C_17 : 0_Cyclo	1.7	tr
C_18 : 0_	1.1	3.0
C_18 : 1_*ω*9*c*	2.5	1.1
C_18 : 0_ 2OH	1.5	tr
Summed feature 3*	39.7	41.2
Summed feature 8†	17.2	11.4

*Summed In Ffeature 3 = C_16 : 1_ω*7c* and/or C_16 : 1_ω6*c*.

†Summed In Ffeature 8 = C_18 : 1_ω*7c* and/or C_18 : 1_ω6*c*.

In summary, as indicated by the phylogenetic trees, MAHUQ-54^T^ represents a member of the genus *Aquincola*. In addition, the characteristics of MAHUQ-54^T^ are consistent with descriptions of the members of the genus *Aquincola* with regard to morphological, biochemical and chemotaxonomic properties. However, MAHUQ-54^T^ can be distinguished from the most closely related type strain not only by physiological and biochemical characteristics but also by low dDDH values and ANI values. The results of this polyphasic comparison between MAHUQ-54^T^ and its close phylogenetic neighbours indicated thatMAHUQ-54^T^ should be assigned to the genus *Aquincola* as the type strain of a novel species, for which the name *Aquincola agrisoli* sp. nov. is proposed.

## Description of *Aquincola agrisoli* sp. nov.

*Aquincola agrisoli* (a.gri.so′li. L. masc. n. *ager*, field, farm; L. neut. n. s*olum*, soil; N.L. gen. neut. n. *agrisoli*, of or belonging to farmland soil).

Cells are Gram-stain-negative, aerobic, motile, flagellated and rod-shaped (0.5–1.1 µm width and 1.2–2.7 µm long). Colonies on R2A agar medium are smooth, spherical, light pink coloured and 0.2–0.6 mm in diameter when grown for 2 days. Positive results are obtained for both catalase and oxidase tests. Flexirubin-type pigments are absent. Growth occurs on TSA, NA, LB agar and R2A agar. Growth occurs at 15–40 °C (optimum 30–35 °C) and at pH 5.5–9.0 (optimum 7.0). In R2A broth medium, growth occurs in the presence of 0–0.5 % (w/v) NaCl (optimum 0 %). Cells are able to hydrolyse l-tyrosine, Tween 80, Tween 20, l-arginine and urea but not starch, aesculin, casein, gelatin or DNA. The test for nitrate reduction gives positive results, but indole production and glucose fermentation are negative. Positive for the following enzyme activities: alkaline phosphatase, acid phosphatase, leucine arylamidase, valine arylamidase, cysteine arylamidase, α-chymotrypsin and β-glucuronidase; weakly positive for lipase (C14), trypsin, naphthol-AS-BI-phosphohydrolase, *N*-acetyl-β-glucosaminidase, esterase (C4) and esterase lipase (C8); but negative for α-mannosidase, β-glucosidase, β-galactosidase, α-glucosidase, α-fucosidase and α-galactosidase (API ZYM). d-mannitol, gluconate, adipic acid, malic acid and phenylacetic acid are utilised as sole carbon sources, but the following compounds are not utilised as sole carbon sources: d-glucose, l-arabinose, *N*-acetyl-glucosamine, d-mannose, maltose, capric acid and triosodium citrate (API 20NE). The predominant isoprenoid quinone is Q-8. The major fatty acids are C_16  :  0_, summed feature 3 (comprising C_16  :  1_ω7*c* and/or C_16  :  1_ω6*c*) and summed feature 8 (comprising C_18  :  1_ω7*c* and/or C_18  :  1_ω6*c*).

The type strain, MAHUQ-54^T^ (=KACC 22001^T^ = CGMCC 1.18515^T^), was isolated from the rhizospheric soil of eggplant located in Magura, Bangladesh. The genomic DNA G+C content of the type strain is 70.4 %.The NCBI GenBank accession number for the 16S rRNA gene and draft genome sequences of MAHUQ-54^T^ are MT514502 and JAZIBG000000000, respectively.

## supplementary material

10.1099/ijsem.0.006355 Uncited Supplementary Material 1.

## Abbreviations

ANI: average nucleotide identity
BGCs: biosynthetic gene clusters
dDDH: digital DNA–DNA hybridisation
GBDP: Genome blast Distance Phylogeny
ML: maximum-likelihood
MLSA: multi-locus sequence analysis
MP: maximum-parsimony
NJ: neighbor-joining
RAST: Rapid Annotation using Subsystems Technology
