# Supplementary material for: Aquincola agrisoli sp. nov., isolated from rhizospheric soil of eggplant and in silico genome mining for the prediction of biosynthetic gene clusters
Source: Int J Syst Evol Microbiol. 2024 Apr 29;74(4):006355. doi: 10.1099/ijsem.0.006355 (PMC11092185; doi:10.1099/ijsem.0.006355)
Supplement: Uncited Supplementary Material 1. [file ijsem-74-06355-s001.pdf]

Supplementary Material

Aquincola agrisoli sp. nov., isolated from rhizospheric soil of eggplant and in silico genome mining for the prediction of biosynthetic gene clusters.

Md. Amdadul Huq[1*], Md. Shahedur Rahman[2], M. Mizanur Rahman[3].

[1] *Department of Food and Nutrition, College of Biotechnology and Natural Resource, Chung-Ang University, Anseong-si, Gyeonggi-do, 17546, Republic of Korea.*

[2] *Department of Genetic Engineering and Biotechnology, Jashore University of Science and Technology, Jashore 7408, Bangladesh.*

[3] *Department of Biotechnology and Genetic Engineering, Faculty of Biological Science, Islamic University, Kushtia-7003, Bangladesh.*

[*]Corresponding author:

Md. Amdadul Huq, E-mail: amdadbge@gmail.com, amdadbge100@cau.ac.kr

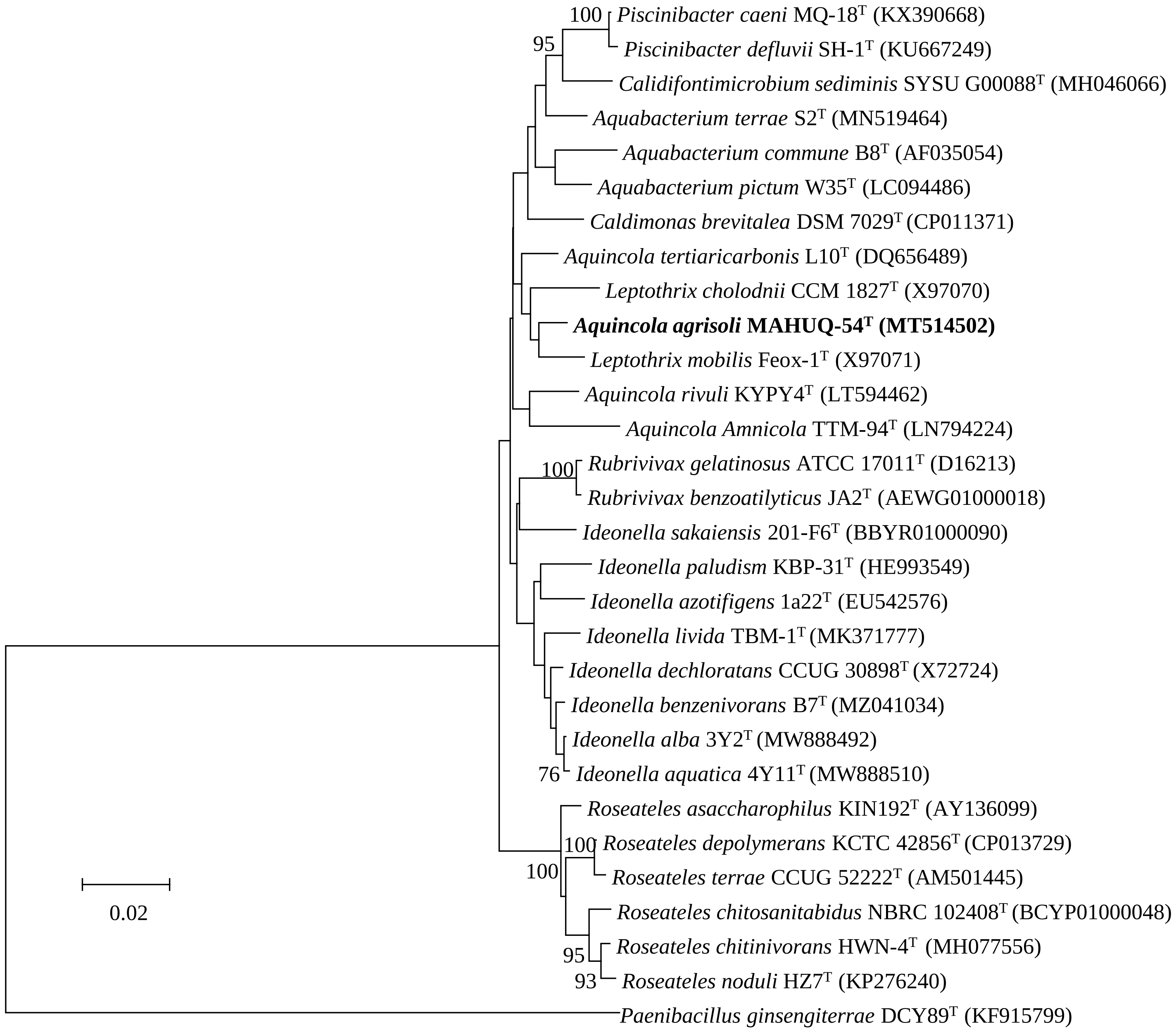

Supplementary Fig. S1. Neighbor-joining phylogenetic tree based on 16S rRNA gene sequences showing the position of *Aquincola agrisoli* MAHUQ-54^T and other related species. Bootstrap values more than 70 % based on 1,000 replications are shown at branching points. *Paenibacillus ginsengiterrae* DCY89^T was used as an outgroup. Scale bar, 0.02 substitutions per nucleotide position.

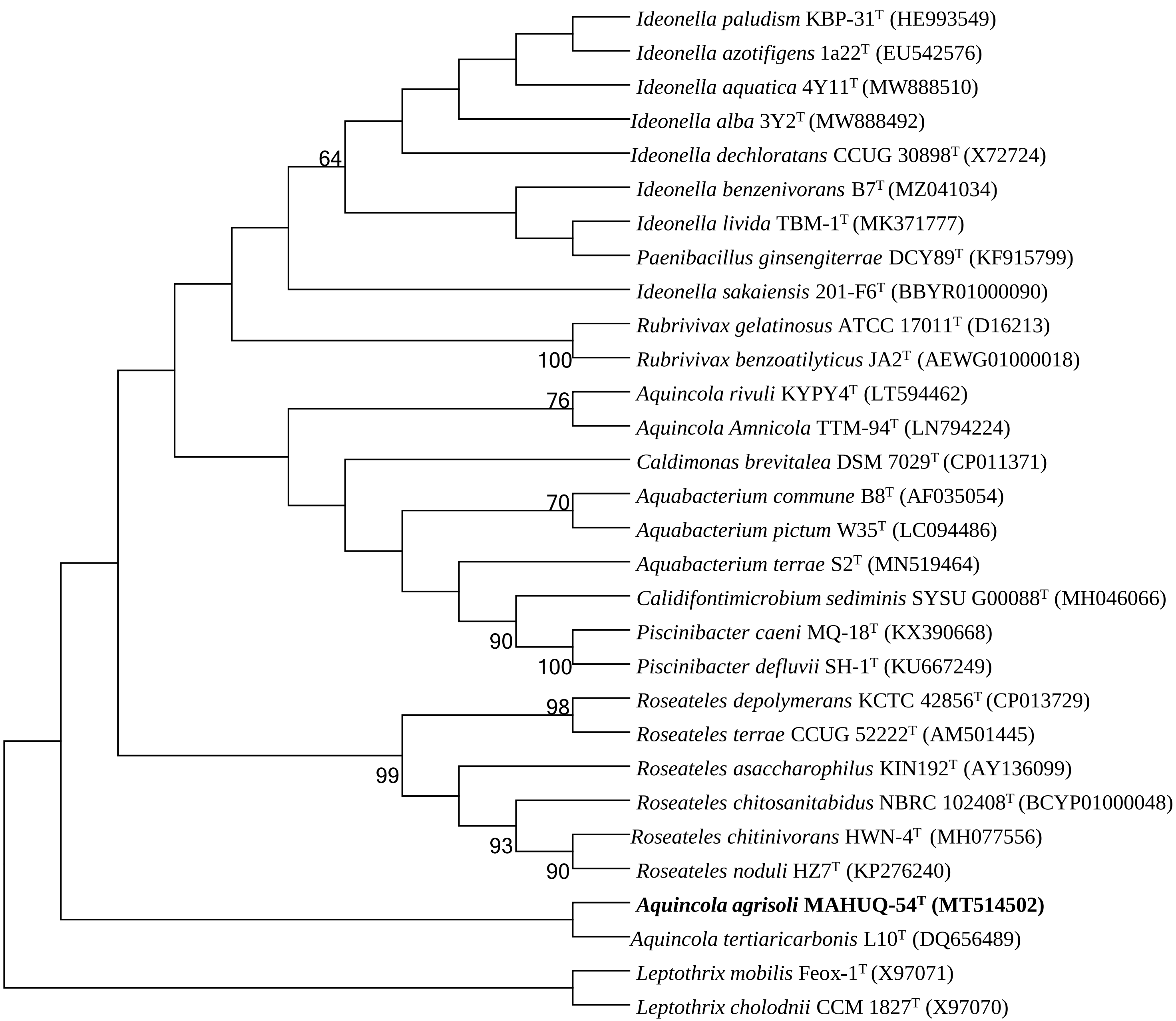

Supplementary Fig. S2. The maximum-parsimony (MP) tree based on 16S rRNA gene sequence analysis showing the position of *Aquincola agrisoli* MAHUQ-54^{T} and other related species. Values less than 60 % were not shown.

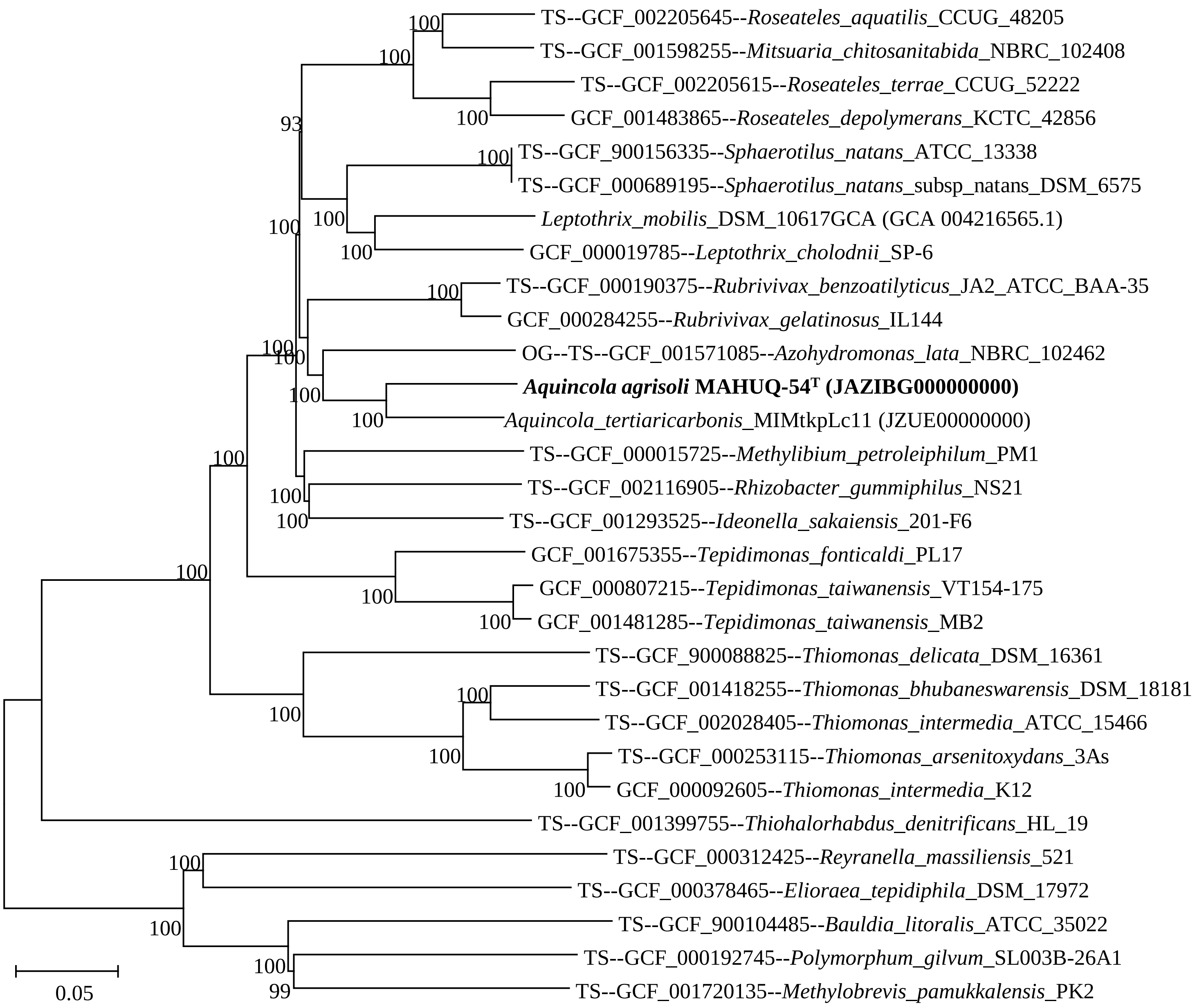

Supplementary Fig. S3. Phylogenetic tree constructed from a comparative analysis of whole genome sequences showing the relationships of strain MAHUQ-54^{T} with other closest species. This tree was constructed via Automated Multi-Locus Species Tree online web server, and with Mega-7 program using the aligned sequences of Automated Multi-Locus Species analysis. Bootstrap values (expressed as percentages of 1000 replications) greater than 50 % are shown at the branch points. The Bar represents 0.05 substitutions per nucleotide position.

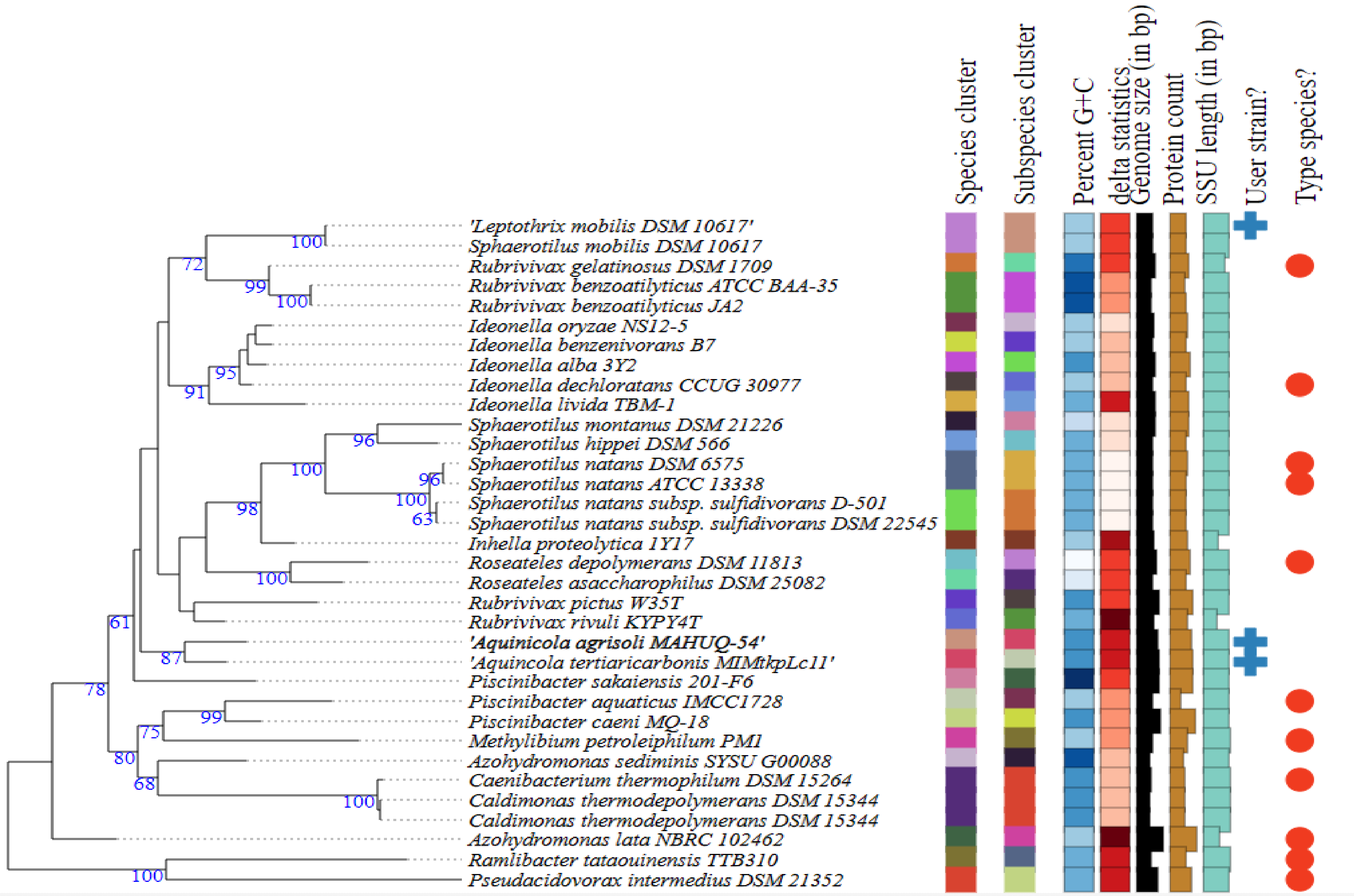

Supplementary Fig. S4. 16s rRNA gene sequence-based GBDP (Genome BLAST Distance Phylogeny) tree showing the relationships of strain MAHUQ-54^{T} with other closest type species. Tree inferred with FastME 2.1.6.1 from GBDP distances calculated from 16s rRNA gene sequences. The branch lengths are scaled in terms of GBDP distance formula d5. The numbers above branches are GBDP pseudo-bootstrap support values > 60 % from 100 replications, with an average branch support of 74.7 %. The tree was rooted at the midpoint.

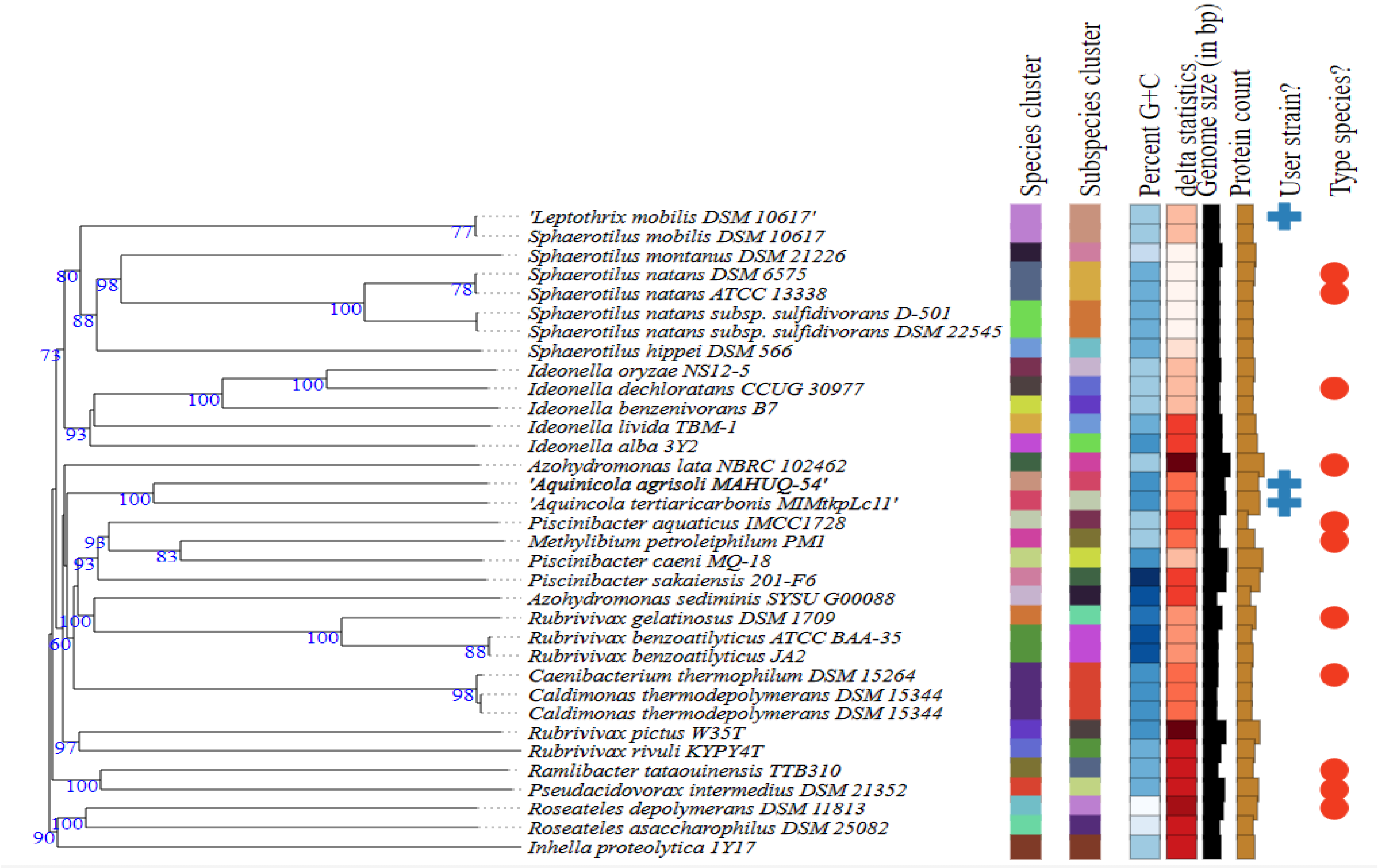

Supplementary Fig. S5. Whole-genome sequence-based GBDP (Genome BLAST Distance Phylogeny) tree showing the relationships of strain MAHUQ-54^{T} with other closest type species. Tree inferred with FastME 2.1.6.1 from GBDP distances calculated from genome sequences. The branch lengths are scaled in terms of GBDP distance formula d5. The numbers above branches are GBDP pseudo-bootstrap support values > 60 % from 100 replications, with an average branch support of 79.1 %. The tree was rooted at the midpoint.

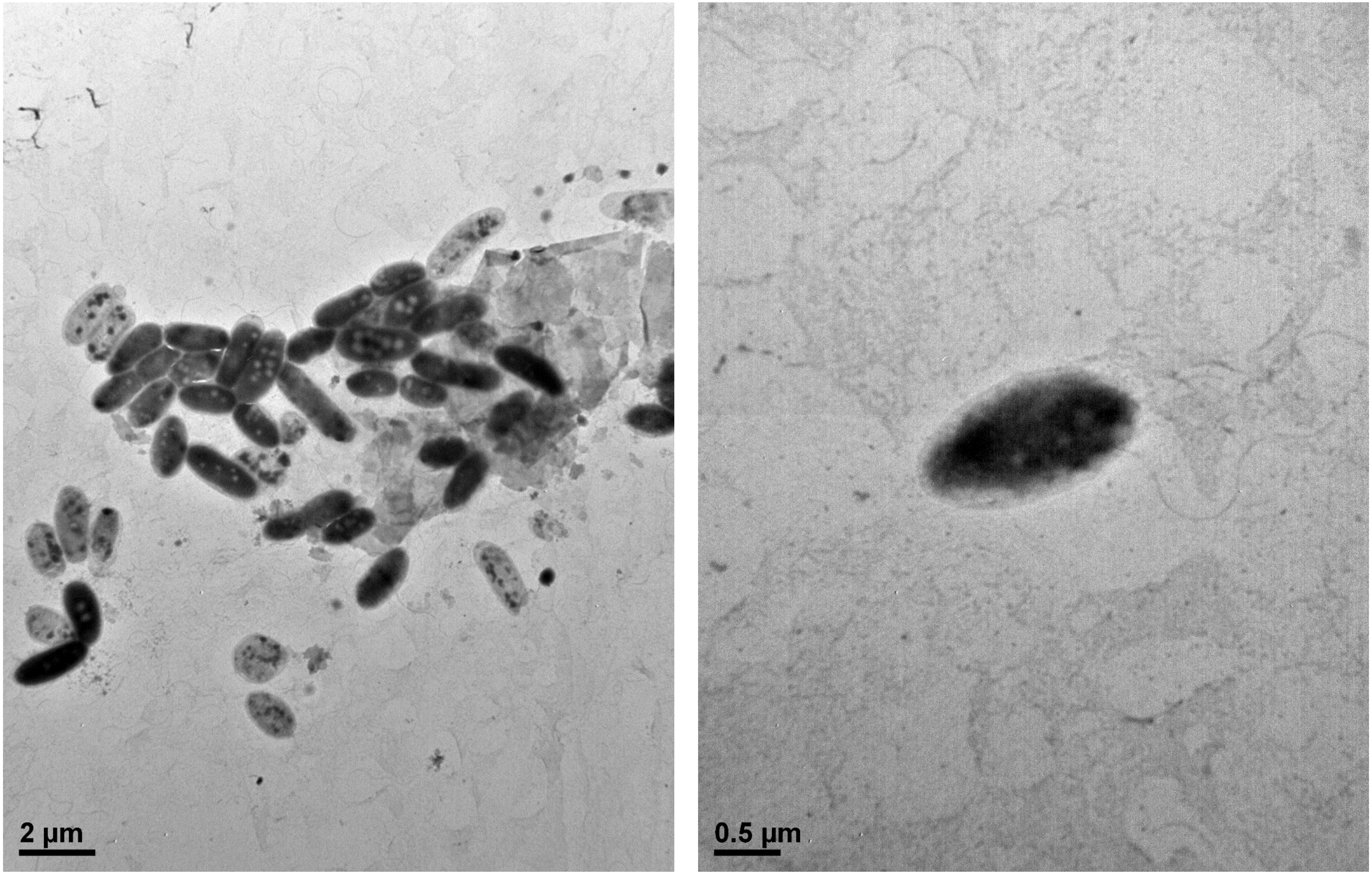

Supplementary Fig. S6. Transmission electron micrograph of cells of *Aquincola agrisoli* MAHUQ-54^{T} after negative staining with uranyl acetate.

Supplementary Table S1. Genome sequence features of novel strain *Aquincola agrisoli* MAHUQ-54^T.

**Features**	***Aquincola agrisoli* MAHUQ-54^T**
Accession No.	JAZIBG000000000
Biosample	SAMN39849881
BioProject	PRJNA1074115
Total sequence length (nt)	5,994,516
Scaffold N50	359,003
Scaffold N75	166,781
Number of contigs	60
Sequencing method	de novo (illumina XTen)
Annotation pipeline	NCBI Prokaryotic Genome
DNA G+C content (%)	70.4
Total genes	5,428
Genes (coding)	5,348
Number of RNAs	54
tRNAs	45
rRNAs	5

Supplementary Table S2. dDDH and ANI values between the proposed novel strain *Aquincola agrisoli* MAHUQ-54^{T} and the closest type strains.

**Query genome**	**Reference genome**	**dDDH value**				**ANI (%)**
		DDH	Model C.I.	Distance	Prob. DDH >= 70%	
Aquincola agrisoli MAHUQ-54^{T} (JAZIBG000000000)	*Aquincola tertiaricarbonis* MIMtkpLc11 (JZUE00000000)	25.3%	[23 - 27.8%]	0.1718	0.01%	83.35
	Leptothrix mobilis DSM 10 (GCA 004216565.1)	20.8%	[18.5 - 23.2%]	0.2116	0%	75.89

Supplementary Table S3. Pairwise comparisons of user genome (*Aquinicola agrisoli* MAHUQ-54^{T}) vs type strain genomes.

Type Strain Genome Server								
**Query strain**	**Subject strain**	**dDDH (d0, in %)**	**C.I. (d0, in %)**	**dDDH (d4, in %)**	**C.I. (d4, in %)**	**dDDH (d6, in %)**	**C.I. (d6, in %)**	**G+C content difference (in %)**
Aquinicola agrisoli MAHUQ-54	*Aquincola tertiaricarbonis* MIMtkpLc11	35	[31.6 - 38.5]	25.3	[23.0 - 27.8]	31.6	[28.7 - 34.7]	0.19
Aquinicola agrisoli MAHUQ-54	*Azohydromonas lata* NBRC 102462	16.8	[13.8 - 20.3]	21	[18.7 - 23.4]	16.7	[14.1 - 19.7]	1.39
Aquinicola agrisoli MAHUQ-54	*Piscinibacter caeni* MQ-18	19.5	[16.3 - 23.1]	20.9	[18.7 - 23.4]	18.9	[16.2 - 21.9]	0.21
Aquinicola agrisoli MAHUQ-54	*Piscinibacter sakaiensis* 201-F6	18.6	[15.5 - 22.2]	20.9	[18.7 - 23.3]	18.2	[15.5 - 21.2]	2.54
Aquinicola agrisoli MAHUQ-54	*Caldimonas thermodepolymerans* DSM 15344	19	[15.9 - 22.6]	20.8	[18.6 - 23.3]	18.5	[15.8 - 21.5]	0.06
Aquinicola agrisoli MAHUQ-54	*Rubrivivax benzoatilyticus* JA2	19.6	[16.4 - 23.2]	20.8	[18.5 - 23.2]	19	[16.3 - 22.0]	1.22
Aquinicola agrisoli MAHUQ-54	*Sphaerotilus mobilis* DSM 10617	16.8	[13.8 - 20.3]	20.8	[18.5 - 23.2]	16.7	[14.1 - 19.6]	1.33
Aquinicola agrisoli MAHUQ-54	*Azohydromonas sediminis* SYSU G00088	19.9	[16.7 - 23.5]	20.8	[18.6 - 23.2]	19.2	[16.5 - 22.2]	1.46
Aquinicola agrisoli MAHUQ-54	*Caenibacterium thermophilum* DSM 15264	18.9	[15.7 - 22.4]	20.8	[18.6 - 23.2]	18.4	[15.7 - 21.4]	0.12
Aquinicola agrisoli MAHUQ-54	'*Leptothrix mobilis* DSM 10617'	16.8	[13.8 - 20.3]	20.8	[18.5 - 23.2]	16.7	[14.1 - 19.6]	1.37
Aquinicola agrisoli MAHUQ-54	*Sphaerotilus natans* subsp. sulfidivorans D-501	17.7	[14.6 - 21.2]	20.7	[18.5 - 23.1]	17.4	[14.8 - 20.4]	0.47
Aquinicola agrisoli MAHUQ-54	*Methylibium petroleiphilum* PM1	18.6	[15.4 - 22.1]	20.7	[18.5 - 23.1]	18.1	[15.5 - 21.1]	1.59
Aquinicola agrisoli MAHUQ-54	*Sphaerotilus nata*ns DSM 6575	17.6	[14.6 - 21.2]	20.7	[18.5 - 23.1]	17.3	[14.7 - 20.3]	0.42
Aquinicola agrisoli MAHUQ-54	*Sphaerotilus natans* ATCC 13338	17.7	[14.6 - 21.2]	20.7	[18.5 - 23.1]	17.4	[14.8 - 20.4]	0.43
Aquinicola agrisoli MAHUQ-54	*Piscinibacter aquaticus* IMCC1728	20.3	[17.1 - 23.9]	20.7	[18.5 - 23.1]	19.5	[16.8 - 22.5]	1
Aquinicola agrisoli MAHUQ-54	*Rubrivivax benzoatilyticus* BAA-35	19.7	[16.5 - 23.3]	20.7	[18.5 - 23.1]	19	[16.3 - 22.0]	1.16
Aquinicola agrisoli MAHUQ-54	*Roseateles depolymerans* DSM 11813	15	[12.2 - 18.5]	20.6	[18.4 - 23.0]	15.2	[12.7 - 18.0]	3.76
Aquinicola agrisoli MAHUQ-54	*Sphaerotilus hippei* DSM 566	17.7	[14.7 - 21.3]	20.6	[18.4 - 23.0]	17.4	[14.8 - 20.4]	0.34
Aquinicola agrisoli MAHUQ-54	*Ideonella alba* 3Y2	18.1	[15.0 - 21.7]	20.5	[18.3 - 22.9]	17.7	[15.1 - 20.7]	0.17
Aquinicola agrisoli MAHUQ-54	*Ideonella livida* TBM-1	15.6	[12.7 - 19.1]	20.4	[18.2 - 22.9]	15.7	[13.2 - 18.6]	0.36
Aquinicola agrisoli MAHUQ-54	*Rubrivivax pictus* W35T	18.7	[15.6 - 22.3]	20.4	[18.2 - 22.8]	18.2	[15.5 - 21.2]	0.11
Aquinicola agrisoli MAHUQ-54	*Ideonella dechloratans* CCUG 30977	17.3	[14.2 - 20.8]	20.4	[18.2 - 22.8]	17	[14.4 - 20.0]	1.09
Aquinicola agrisoli MAHUQ-54	*Rubrivivax gelatinosus* DSM 1709	19.5	[16.3 - 23.1]	20.3	[18.1 - 22.7]	18.8	[16.2 - 21.9]	1.08
Aquinicola agrisoli MAHUQ-54	*Sphaerotilus montanus* DSM 21226	17.3	[14.3 - 20.8]	20.3	[18.1 - 22.7]	17.1	[14.5 - 20.0]	2.21
Aquinicola agrisoli MAHUQ-54	*Roseateles asaccharophilus* DSM 25082	16.1	[13.1 - 19.6]	20.2	[18.0 - 22.6]	16.1	[13.5 - 19.0]	2.84
Aquinicola agrisoli MAHUQ-54	*Ideonella benzenivorans* B7	17.1	[14.0 - 20.6]	20.2	[17.9 - 22.6]	16.9	[14.3 - 19.8]	1.55
Aquinicola agrisoli MAHUQ-54	*Ideonella oryzae* NS12-5	17.2	[14.2 - 20.8]	20.1	[17.9 - 22.5]	17	[14.4 - 20.0]	1.34
Aquinicola agrisoli MAHUQ-54	*Pseudacidovorax intermedius* DSM 21352	16.5	[13.5 - 20.0]	20	[17.8 - 22.4]	16.3	[13.8 - 19.3]	0.93
Aquinicola agrisoli MAHUQ-54	*Ramlibacter tataouinensis* TTB310	16.4	[13.4 - 19.9]	20	[17.8 - 22.4]	16.3	[13.8 - 19.2]	0.37
Aquinicola agrisoli MAHUQ-54	*Rubrivivax rivuli* KYPY4T	18.1	[15.0 - 21.6]	19.9	[17.7 - 22.3]	17.7	[15.0 - 20.6]	0.3
Aquinicola agrisoli MAHUQ-54	*Inhella proteolytica* 1Y17	15.5	[12.5 - 18.9]	19.3	[17.1 - 21.7]	15.5	[13.0 - 18.4]	1.07

Supplementary Table S4. Distribution of genes based on COG functional categories in the genomes of novel strain *Aquincola agrisoli* MAHUQ-54^T and the closest type strain *Aquincola tertiaricarbonis* L10^T.

**COG Function**	***Aquincola agrisoli***	***Aquincola tertiaricarbonis***
Cofactors, vitamins, prosthetic groups, pigments	187	156
Cell wall and capsule	27	32
Virulence, disease and defense	39	36
Potassium metabolism	12	13
Photosynthesis	0	8
Miscellaneous	33	25
Phages, prophages, transposable elements, plasmids	2	3
Membrane transport	118	116
Iron acquisition and metabolism	10	5
RNA metabolism	52	43
Nucleosides and nucleotides	94	93
Protein metabolism	201	186
Cell division and cell cycle	0	0
Motility and chemotaxis	36	40
Regulation and cell signaling	36	33
Secondary metabolism	5	5
DNA metabolism	82	84
Fatty acids, lipids, and isoprenoids	107	95
Nitrogen metabolism	26	12
Dormancy and sporulation	1	1
Respiration	116	103
Stress response	81	79
Metabolism of aromatic compounds	86	63
Amino acids and derivatives	337	381
Sulfur metabolism	19	8
Phosphorus metabolism	28	25
Carbohydrates	280	265
